# Prospective external validation of an accelerated (2-h) acute coronary syndrome rule-out process using a contemporary troponin assay

**DOI:** 10.1186/s12245-014-0042-3

**Published:** 2014-10-16

**Authors:** Anne-Maree Kelly, Sharon Klim

**Affiliations:** 1 Joseph Epstein Centre for Emergency Medicine Research at Western Health, Sunshine Hospital, Furlong Road, St Albans 3021, Australia; 2 School of Public Health, Queensland University of Technology, Victoria Park Road, , Brisbane, Kelvin Grove, Australia

**Keywords:** Chest pain, Troponin, Acute coronary syndrome

## Abstract

**Background:**

Recently there have been efforts to derive safe, efficient processes to rule out acute coronary syndrome (ACS) in emergency department (ED) chest pain patients. We aimed to prospectively validate an ACS assessment pathway (the 2-Hour Accelerated Diagnostic Protocol to Assess Patients with Chest Pain Symptoms Using Contemporary Troponins as the Only Biomarker (ADAPT) pathway) under pragmatic ED working conditions.

**Methods:**

This prospective cohort study included patients with atraumatic chest pain in whom ACS was suspected but who did not have clear evidence of ischaemia on ECG. Thrombolysis in myocardial infarction (TIMI) score and troponin (TnI Ultra) were measured at ED presentation, 2 h later and according to current national recommendations. The primary outcome of interest was the occurrence of major adverse cardiac events (MACE) including prevalent myocardial infarction (MI) at 30 days in the group who had a TIMI score of 0 and had presentation and 2-h TnI assays <99th percentile.

**Results:**

Eight hundred and forty patients were studied of whom 177 (21%) had a TIMI score of 0. There were no MI, MACE or revascularization in the per protocol and intention-to-treat 2-h troponin groups (0%, 95% confidence interval (CI) 0% to 4.5% and 0%, 95% CI 0% to 3.8%, respectively). The negative predictive value (NPV) was 100% (95% CI 95.5% to 100%) and 100% (95% CI 96.2% to 100%), respectively.

**Conclusions:**

A 2-h accelerated rule-out process for ED chest pain patients using electrocardiography, a TIMI score of 0 and a contemporary sensitive troponin assay accurately identifies a group at very low risk of 30-day MI or MACE.

## 1 Background

Chest pain is a common reason for presentation to emergency departments (ED) accounting for approximately 2.4% of all ED attendances [1]. Between 75% and 85% of patients are ruled out for acute coronary syndrome (ACS) [2]. International guidelines for the investigation of ACS recommend serial measurements of contemporary (non-high sensitivity) cardiac troponin over 4 to 12 h from symptom onset or presentation to the ED [3]-[6]. This can contribute to ED overcrowding with resulting adverse patient outcomes, including increased mortality, and increased costs [7].

Over recent years, there have been efforts to derive safe and efficient assessment processes - in particular attempts to define a subgroup of patients at very low risk and suitable for an accelerated assessment and early discharge. One such approach has been combining electrocardiography (ECG), thrombolysis in myocardial infarction (TIMI) score and biomarkers to define a low-risk group suitable for an accelerated pathway [8],[9]. Such approaches would classify 10% to 20% of chest pain presentations as low risk [8],[9] and have reported negative predictive values exceeding 99%. To date, this approach has had limited external validation - a retrospective validation using data from the Advantageous Predictors of Acute Coronary Syndromes Evaluation (APACE) trial and a study exploring impact on time to discharge [10],[11].

The aim of this study was to prospectively evaluate the 2-Hour Accelerated Diagnostic Protocol to Assess Patients with Chest Pain Symptoms Using Contemporary Troponins as the Only Biomarker (ADAPT) [9] assessment pathway in an independent ED setting under pragmatic ED working conditions.

## 2 Methods

This prospective cohort study was conducted in the ED of a community teaching hospital with an annual adult ED census of approximately 36,000 between 16 April 2012 and 3 February 2013.

Patients were screened for inclusion if they presented with chest pain. Exclusion criteria were chest pain due to trauma, aged <18 years, no chest pain within 24 h of the index ED visit, chest pain lasting <10 min, no ECG or no troponin assay performed within 24 h of index ED visit, a clear alternative diagnosis at initial medical officer assessment, ischaemic ECG changes at ED presentation, haemodynamic instability, advanced terminal disease, inability to communicate in English and declined/ unavailable for follow-up. We had data collection support 18 h a day (8 am to midnight) so patients presenting outside these hours were also excluded.

The intervention was measurement of troponin 2 h after the sample taken at ED presentation in addition to the ‘routine’ samples taken at ED arrival and at least 3 to 4 h later or 6 h from symptom onset in accordance with current National Heart Foundation (Australia) (NHF) guidelines [5]. While the treating staff were not formally blinded to the result of the 2-h test, it was not used for disposition decision-making. The troponin assay used by the laboratory was TnI-Ultra (Siemens Diagnostics, Munich, Germany) performed on an Advia Centaur analyzer. The test has a reported range of 0.006 to 50 μg/l. Coefficients of variation are 10% at TnI 0.03 μg/l, 5.3% at 0.08 μg/l and 4.1% at 0.18 μg/l. The 99th percentile is 0.04 μg/l (95% confidence interval (CI) 0.03 to 0.05 μg/l) (manufacturer's information).

Data collected included demographics, cardiac risk factors, biomarker assay results, ED disposition, final diagnosis, data to calculate Global Risk Acute Coronary Events Registry (GRACE) risk, freedom-from-events and TIMI scores and 7- and 30-day outcomes. Seven and 30-day outcomes were assessed by the review of medical records and structured telephone follow-up. An independent cardiologist adjudicated on the final diagnosis and outcome for the subgroups where patients with troponin elevations on any test exceeded the 99th percentile and were coded as non-ACS and for patients without troponin elevations who were coded as ACS.

The primary outcome of interest was the occurrence of major adverse cardiac events (MACE) within 30 days in patients who had a TIMI score of 0, had presentation and 2-h TnI <99th percentile. MACE was defined as prevalent (present at index ED visit) or incident (occurring after initial ED visit) myocardial infarction (MI), death, survived cardiac arrest, cardiogenic shock, life-threatening arrhythmia, high-degree atrioventricular block and new atrial fibrillation. These mirror adverse events reported in similar studies. Final diagnosis was assigned by treating cardiologists unaware of the study. The secondary outcome of interest was MACE or revascularization at 30 days in the same group.

Analysis was by descriptive statistics. It became clear that there had been some variability in collection of the 2-h specimens, due largely to competing workload and variations in laboratory time stamp practices. We chose to analyze three groups - a per protocol group where the laboratory time stamps for the initial and 2-h specimens are 2 h ±15 min apart, an intention to treat group where a specimen was time-stamped within the per protocol definition or up to 2 h ±60 min from initial specimen time stamp as long as a further assay at/ after the NHF recommended interval was also taken and a group who did not undergo a 2-h troponin assay (missed group). The missed group underwent biomarker assays at NHF guideline-recommended intervals or longer. Regarding sample size, we had planned to collect data on 150 patients with a 2-h assay (giving a confidence interval ±2%); however, the number of missed patients was higher than anticipated and the study was stopped as data collection support was no longer available.

The study was approved by the institutional ethics panel and was registered with the Australia and New Zealand Clinical Trials Registry (ACTRN12612000990820). Patients provided verbal consent to telephone follow-up.

## 3 Results

**Figure 1:**
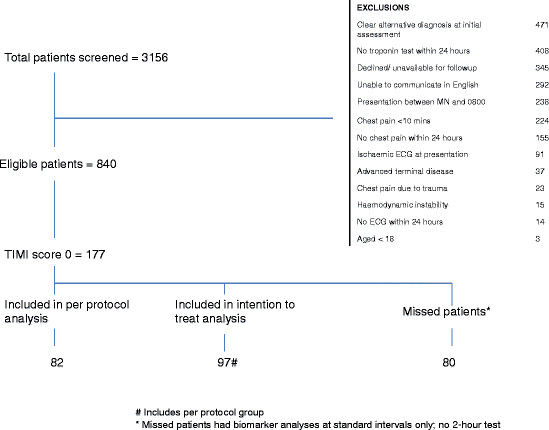
Sample derivation.

Sample derivation is shown in Figure 1. In the 840 eligible patients, 72 had an ACS final diagnosis (13%), 177 (21%) had a TIMI score of 0 and 82 patients fit the criteria for per protocol analysis with a further 15 meeting the criteria for intention-to-treat analysis. Patient characteristics and outcome are summarized in Table 1.

**Table 1:** Patient characteristics and outcome

**Parameter**	**Per protocol group (*N*** **= 82)**	**ITT group (*N*** **= 97)**^**a**^	**Missed patients**^**b**^**(*N*** **= 80)**
Age (median, IQR)	47 (40 to 55)	48 (40 to 55)	49 (41 to 56)
Gender (*N*, %)	43, 52%	53, 55%	36, 46%
Arrival by ambulance (*N*, %)	59, 72%	70, 72%	62, 79%
Killip class 1 (*N*, %)	81, 99%	95, 98%	76, 96%
History of hypertension (*N*, %)	17, 21%	20, 21%	16, 20%
History of diabetes (*N*, %)	5, 6%	5, 5%	2, 3%
History of elevated cholesterol/cholesterol treatment (*N*, %)	16, 20%	18, 19%	21, 27%
Smoker (*N*, %)	27, 33%	32, 33%	16, 20%
Family history CAD (*N*, %)	22, 27%	28, 29%	22, 28%
Hospital admission (*N*, %)	3, 4%	5, 5%	6, 8%
GRACE risk score (median, IQR)	70 (60 to 88)	70 (59 to 88)	77 (60 to 89)
GRACE freedom from events score (median, IQR)	323 (312 to 333)	323 (311 to 333)	325 (312 to 336)
MACE at 7 days (*N*, %, 95% CI)	0, 0%, 0% to 4.5%	0, 0%, 0% to 3.8%	0, 0%, 0% to 4.6%
MACE at 30 days (*N*, %, 95% CI)	0, 0%, 0% to 4.5%	0, 0%, 0% to 3.8%	0, 0%, 0% to 4.6%
MACE or revascularization at 30 days (*N*, %, 95% CI)	0, 0%, 0% to 4.5%	0, 0%, 0% to 3.8%	1, 1.3%, 0.2% to 6.8%

ITT, intention to treat; IQR, interquartile range; CAD, coronary artery disease; GRACE, Global Risk Acute Coronary Events Registry; MACE, major adverse cardiac events. ^a^Includes per protocol group; ^b^standard biomarker interval only, no 2-h specimen.

There were no MI, MACE or revascularization in the per protocol and intention-to-treat 2-h troponin groups (0%, 95% CI 0% to 4.5% and 0%, 95% CI 0% to 3.8%, respectively). The negative predictive value (NPV) was 100% (95% CI 95.5% to 100%) and 100% (95% CI 96.2% to 100%), respectively.

Of note, no included TIMI-0 patient with initial TnI <99th percentile had a biomarker rise at 2 h or the standard testing interval.

## 4 Discussion

An accelerated ACS rule-out process integrating ECG, score-based risk assessment and biomarker assays was first proposed by Than et al. in the Asia-Pacific Evaluation of Chest Pain Trial (ASPECT) study [8]. That international study used a point-of-care multi-biomarker panel and clinical risk stratification using the TIMI score and reported a NPV of 99.1% (95% CI 97.3% to 99.8%). A subsequent analysis of an Australasian cohort largely but not completely overlapping with the ASPECT cohort explored TIMI score 0, normal ECG and normal troponin I using Beckmann Coulter AccuTnI in Australian patients and Abbott Architect cTnI in New Zealand patients (the ADAPT study) [9]. It reported a NPV of 99.7% (95% CI 98.6% to 100%). A further analysis using ADAPT study patients, TIMI 0 and a high-sensitivity troponin assay (Abbott Architect high-sensitivity TnI) reported a NPV of 100% (95% CI 98.8% to 100%) [12]. While the NPVs are impressive, these analyses use data from significantly overlapping cohorts and as such are not external validations. A retrospective validation was performed on the APACE study cohort and reported a NPV of 100% (98.4% to 100%) [12]. In a study exploring the impact of the accelerated pathway on discharge within 6 h, Than reported a NPV of 98.9% (95% CI 94.2% to 99.8%) [13].

Our prospective study adds to the body of evidence that patients with a TIMI score of 0 can have ACS ruled out by an accelerated 2-h rule-out process using a contemporary sensitive troponin assay. Pooled with the results of other studies using the accelerated pathway and sensitive (but non-high sensitive) troponin [9],[11] yields a NPV for MACE at 30 days of 99.7% (95% CI 98.7% to 99.9%). This low level of risk is likely to be acceptable to patients and clinicians. In a previous study, when implementation was studied, the accelerated process resulted in a higher proportion of patients being safely discharged within 6 h of ED presentation (19.3% vs. 11%) [11]. A change of this magnitude if replicated outside of trial conditions would result in clinical meaningful improvements in ED capacity and reduce overcrowding.

Other approaches to accelerated ACS rule out are also being developed. These include modifying the process used in this study to include patients with TIMI score ≤1 and using a high-sensitivity troponin assay [12]. That approach reports NPV in excess of 99% and would classify approximately 40% of patients as low risk and suitable for the accelerated assessment pathway but waits external validation. Other approaches to clinical risk assessment such as the EDACS score [13], the HEART score [14] and the North American Chest pain rule [15] have been developed, and their use in combination with ECG and biomarkers is under investigation. Yet another approach being explored is the use of changes in troponin level over time (delta troponin) [16]. Which approach or approaches prove to be practical, reproducible, safe and effective and whether any are superior to the approach studied here will evolve over the next few years.

In this study, we found that no included patients with a TIMI score of 0 and initial TnI <99th percentile went on to have a significant troponin rise or MI diagnosis yielding a NPV of 100% (95% CI 97.9% to 100%). This study was not designed or powered to test this hypothesis so it would be inappropriate to draw conclusions from this result. That said, it may be worthy of further study.

This study has some limitations that should be considered when interpreting its results. It was a single-centre study; so, it may not be generalizable to other sites, although its results are consistent with those of other centres. The sample size is less than what was planned resulting in wider than desired confidence intervals. The study was conducted under pragmatic ED conditions so samples for troponin assay were not always collected at the exact planned intervals; however, this is the reality of ED clinical practice. While patients were identified prospectively, some data regarding risk factors, etc. were collected retrospectively so may have been subject to documentation error.

## 5 Conclusions

A 2-h accelerated rule-out process for ED chest pain patients using ECG, TIMI score of 0 and a contemporary sensitive troponin assay accurately identifies a group at low risk of 30-day MI or MACE.

## Competing interests

Professor Kelly was a member of the core writing group of the National Heart Foundation ACS guidelines 2005 to 2014.

## Authors’ contributions

AMK had the concept for the study, contributed to development of the study design and methodology, performed the analysis, contributed to interpretation of the results, drafted the manuscript and contributed to its refinement. SK contributed to development of the study design and methodology, managed data collection and entry, contributed to interpretation of the results and contributed to refinement of the manuscript. Both authors read and approved the final manuscript.
